# Development and clinical application of deep learning model for lung nodules screening on CT images

**DOI:** 10.1038/s41598-020-70629-3

**Published:** 2020-08-12

**Authors:** Sijia Cui, Shuai Ming, Yi Lin, Fanghong Chen, Qiang Shen, Hui Li, Gen Chen, Xiangyang Gong, Haochu Wang

**Affiliations:** 1 Department of Radiology, Zhejiang Provincial People’s Hospital, Affiliated People’s Hospital of Hangzhou Medical College, Hangzhou, 310013 China; 2 The Second Clinical Medical College, Zhejiang Chinese Medical University, Hangzhou, 310053 China; 3 Hangzhou Yitu Healthcare Technology Co., Ltd, Hangzhou, 310000 China; 4 Institute of Artificial Intelligence and Remote Imaging, Hangzhou Medical College, Hangzhou, 310000 China

**Keywords:** Health care, Mathematics and computing

## Abstract

Lung cancer screening based on low-dose CT (LDCT) has now been widely applied because of its effectiveness and ease of performance. Radiologists who evaluate a large LDCT screening images face enormous challenges, including mechanical repetition and boring work, the easy omission of small nodules, lack of consistent criteria, etc. It requires an efficient method for helping radiologists improve nodule detection accuracy with efficiency and cost-effectiveness. Many novel deep neural network-based systems have demonstrated the potential for use in the proposed technique to detect lung nodules. However, the effectiveness of clinical practice has not been fully recognized or proven. Therefore, the aim of this study to develop and assess a deep learning (DL) algorithm in identifying pulmonary nodules (PNs) on LDCT and investigate the prevalence of the PNs in China. Radiologists and algorithm performance were assessed using the FROC score, ROC-AUC, and average time consumption. Agreement between the reference standard and the DL algorithm in detecting positive nodules was assessed per-study by Bland–Altman analysis. The Lung Nodule Analysis (LUNA) public database was used as the external test. The prevalence of NCPNs was investigated as well as other detailed information regarding the number of pulmonary nodules, their location, and characteristics, as interpreted by two radiologists.

## Introduction

Lung cancer screening has now been widely applied because of its effectiveness and ease of performance. More than 10 million chest CT scans were performed in the United States alone in 2012, highlighting the potential for this clinical scenario^1^. Radiologists who evaluate a large number of low-dose CT (LDCT) screening images face enormous challenges, including mechanical repetition and boring work, the easy omission of small nodules, lack of consistent criteria, etc.^2–5^.

Artificial intelligence (AI) is showing rapid advantages and exciting achievements in the fields of imaging diagnosis and/or evaluation^6–12^. AI detection of lung nodules has long been expected to be an effective assistant in daily clinical practice, especially for LDCT lung nodule screening. Thus far, many novel deep neural network-based systems have demonstrated the potential for use in the proposed technique for helping radiologists improve nodule detection accuracy with efficiency and cost-effectiveness^13–18^. However, the majority of the proposed systems were trained on CT scans from the Lung Image Database Consortium/Image Database Resource Initiative (LIDC-IDRI), the LUNA16 (Lung Nodule Analysis 2016) database and the ANODE09 (Automatic Nodule Detection 2009) database^19–24^. The effectiveness in clinical practice has not been fully recognized or proven. From the previous study, the prevalence of pulmonary nodules (PNs) varies greatly in different populations, ranging from 13 to 58%^25–32^. Different demographic features, selection criteria of participants, and the referral pattern of the study centre may explain such differences^27^. In China, the incidence of non-calcified pulmonary nodules (NCPNs) in a large population is rarely reported.

Recent advances in artificial intelligence (AI) have made great strides in automatically quantifying radiographic patterns in medical imaging data. The aim of this study was to assess the performance and effectiveness of deep neural networks for lung nodule detection by comparing the diagnostic efficacy of this AI system with that of radiologists evaluating clinical LDCT cases, in addition, performed a large-scale analysis of lung LDCT screening in three medical centres, investigate the prevalence and characteristics of the NCPNs in Chinese population.

## Materials and method

### Study population and data sets

This retrospective study was approved by the ethics committee of Zhejiang Provincial People’s hospital. The ethics committee approved this study waived the need for informed consent. All methods were performed in accordance with the relevant guidelines and regulations.

A data set of 39,014 chest LDCT screening cases was retrospectively collected from three hospitals between January and June 2017. All examinations were performed as a part of routine healthcare plans. The institutional review committees approved the study. Informed consent was waived due to retrospective use of patient imaging data. The study was registered with www.chictr.org.cn (ChiCTR1800016226).

**Figure 1:**
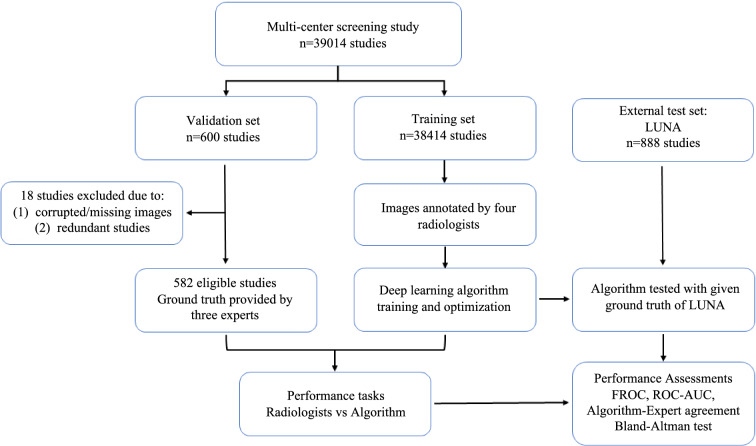
Data flowchart of the study design illustrating development process of a deep neural network-based learning algorithm, which was doubly tested with two datasets: a multi-centre validation set and the LUNA data.

The image set of was divided into two data subsets: a training set of 38,414 cases and a validation set of 600 cases (Fig. 1). There were no overlapping cases between the two data sets. The training set was used to train the model parameters of the pre-trained DL network, which was applied in daily clinical work. The validation set was used to evaluate the DL network performance. The training set included annotated/labelled images that four radiologists (each with more than 5 years of experience reading chest CT scans) independently interpreted and diagnostically evaluated for the presence of lung nodules. To account for inter-rater discrepancy due to human error, any disagreement in the annotation was arbitrated by a senior radiologist with more than 10 years of experience. The validation set included randomly selected chest LDCT cases from the same screening cohort.

To further evaluate the performance of the model relative to the results of our multi-centre study, we used a third data set from the LUNA image collection (Lung Image Database Consortium, https://wiki.cancerimagingarchive.net/display/Public/LIDC-IDRI, and https://luna16.grand-challenge.org/home established by the NIH and NCI of the United States) with available ground truth diagnosis as our external test set. In total, 888 chest CT cases were included in the LUNA data set, and a corresponding performance assessment was also performed.

Another data set of 94,892 chest LDCT scanning cases between Oct. 2017 to Oct. 2018 was retrospectively collected from three medical centers to investigate the prevalence of pulmonary nodules in China. All examinations were performed as a part of routine healthcare plans, including body check-up of the enterprises and institutions or the individual, screening for lung cancer, tuberculosis, pneumoconiosis, etc. Participants with incomplete record of demographic data (n = 46), participants younger than 18 years old (n = 969) were excluded from this study, most of them were for School Entrance Exams and Pre-departure Medical Check, participants with medical history of tuberculosis were excluded from this study (n = 144). Participants with medical history of pulmonary nodules were also excluded from the study (n = 29,565). A total of 64,168 participants were finally enrolled in this study to explore the prevalence of pulmonary nodules in China.

### AI algorithm and image explanation

**Figure 2:**
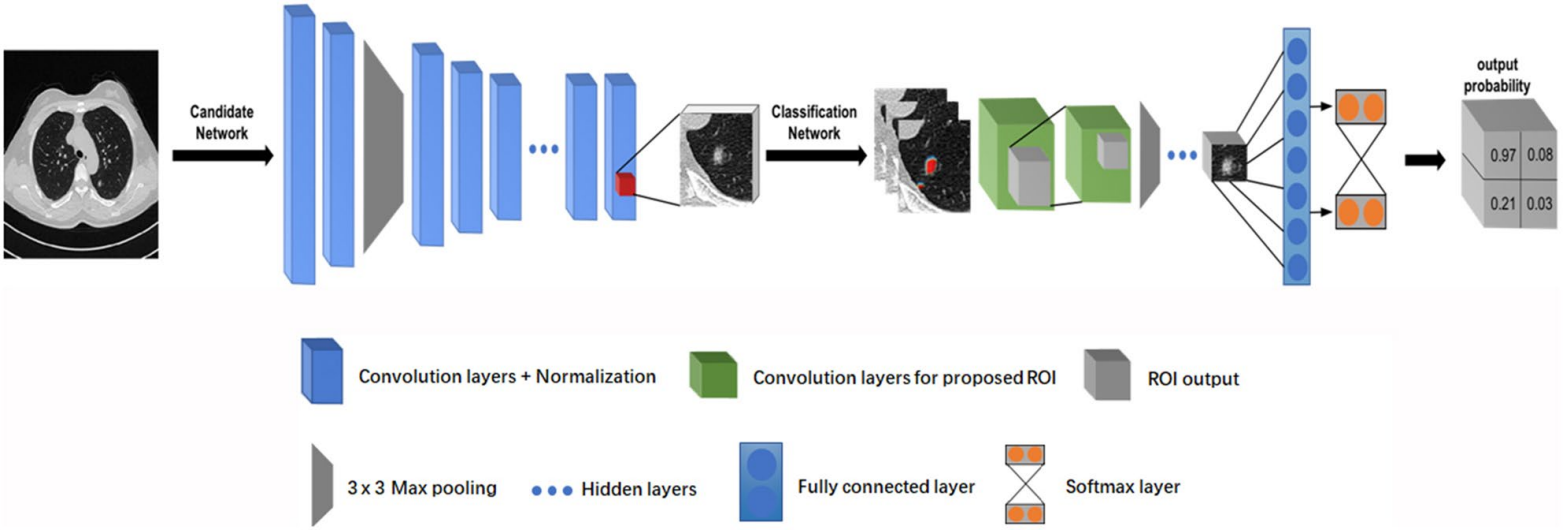
Schematic representation of deep neural network architecture.

**Figure 3:**
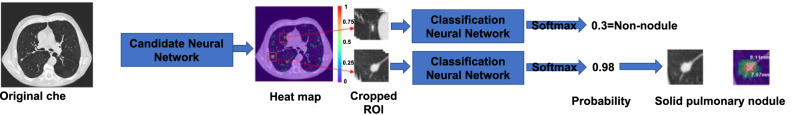
Diagnostic workflow of the two-step modules of the DL algorithm. After analysis by the DL model, the ROI near the right lower lobe on the heat map was confirmed to be a solid pulmonary nodule, and the nodule-like mass in the right upper lobe was eventually confirmed to be a blood vessel.

The algorithm was developed on a convolutional neural architecture known as a deep residual network (ResNet, https://arxiv.org/abs/1512.03385). ResNet uses 50 layers of a deep convolutional neural network (CNN), which contains 3.6 × 10^9^ floating point operations (i.e., FLOPs). Adaptation of the initiated training to our specific tasks was then designed with two-step algorithm modules: identification of candidate nodules and classification of the proposed nodules (Fig. 2). For the candidate identification, all annotated (labelled) CT images were trained using a fully connected network to generate a pixel-wise confidence score for nodule segmentation. Subsequently, the proposed nodules that met or exceeded the threshold of the confidence score were selected for binary classification to determine whether they should be classified as “nodules”. Samples of feature maps and explainable workflows of the two-step modules of the DL algorithm are shown in Fig. 3. In the first step (candidate neural network), the chest CT images were used as input data to generate a heat map for demonstrating a sensitive region of interest (ROI), which could contain sceptical nodules. The second step (classification neural network) then proceeded to crop the proposed ROIs from the preceding step to classify an answer of “true or false” to the following question: “Which one is lung nodule at a high confidence level?” A softmax classifier was applied to the last network layer to calculate the probability of identifying true nodules to reduce false positives (FPs).

### Performance assessment of the algorithm and human reviewers

The diagnostic performance of the DL algorithm was evaluated in the multi-centre validation set and the external test set (LUNA). To compare the performance between radiologists and the algorithm using the validation set, firstly, we recruited three experts (each with more than 20 years of experience) independently graded the images, and they were blinded to the initial clinical assessments and patient information. The ground truth/reference standard for the validation set was determined by an all-agree or two-thirds-agree rationale for each case. Then we recruited nine human reviewers including 5 senior (with more than 10 years of experience) radiologists and 4 junior (with more than 5 years of experience) radiologists from the three hospitals to independently rate the validation set (n = 582). The nine radiologists reviewed all studies and marked suspected lung nodules without time constraints. The performance of the DL algorithm was also assessed with the same validation set.

Subsequently, the nodule readings from both nine human reviewers and the DL algorithm were compared to the reference standard (i.e., ground truth) given by the consensus of the three experts. In addition to the validation set, LUNA data (n = 888) with ground truths were also used for assessing the performance of the DL algorithm.

This study applied two different performance tasks to evaluate diagnostic metrics and agreement between human reviewers and the DL algorithm. In the first task, the identification of highly suspected lung nodules by junior and senior radiologists and the DL algorithm was assessed in the validation and LUNA data sets. The sensitivity of nodule identification over a range of FP rates per study was plotted using free-response receiver operating characteristic (FROC) curves for the DL algorithm with both the multi-centre validation set and the LUNA data. For the validation set, all nodule readings were categorized into two subgroups by size for assessment of detection agreement to the reference standard: transverse diameter < 4 mm (i.e., micro-nodules) and ≥ 4 mm. For the LUNA data, all nodules were categorized into two subgroups by size according to the ground truth: transverse diameter 4–8 mm and > 8 mm, no nodules were smaller than 4 mm in diameter in the LUNA data set. In the second performance task, the classification of nodule-positive cases by the junior and senior radiologists and the DL algorithm was assessed with the validation set and the LUNA set. All classification results were compared to the ground truth or reference standard, and then diagnostic metrics were obtained and evaluated by receiver operating characteristic (ROC) curves. The duration of time the human reviewers or the algorithm required to complete the task was automatically documented in the workstation.

### Pulmonary nodules identification

How a particular lesion was classified as a nodule was determined by a consensus of at least three of the four radiologists as described by Armato et al.^33^ and the Fleischner guidelines^34^. The Fleischner society defines nodules as a “round opacity at least moderately well marginated” measuring less than 3 cm^35^. A nodule not showing a benign pattern of calcification was defined as non-calcified pulmonary nodules (NCPNs) in this study^34^. The visual definition of calcification is that the lesion density is similar to osseous structures on mediastinal windows^36^. NCPNs were further classified as solid nodules(SNs), part-solid nodules (PSNs), and ground glass nodules (GGNs) according to the convention classification proposed by the radiologists from Fleischner Society^34,37,38^. However, the criteria for these nodules’ distinctions are still controversial. We classified them as Van Riel et al.^39^ described, on thin sections with mediastinal (soft-tissue) window settings and a sharp filter, the nodules that are rendered partially invisible can be regarded as part-solid and that any nodule components other than normal vascular or bronchial structures that remain visible on such images are solid. The nodule size was defined as the mean of maximum and minimum diameter in the axial plane^37^.

### Image recognition for the prevalence of pulmonary nodules in China

After the training and validation of the DL algorithm, 64,168 cases were used to investigate the prevalence of NCPNs in China by DL algorithm, all CT images were automatically analyzed by the DL algorithm at first. Then a junior radiologist checked the result given by the DL algorithm and revised the results when necessary. Finally, an experienced radiologist with more than 20 years of experience confirmed the final decision and issued the diagnostic reports.

### Statistical analysis

Data analyses were conducted with the Python library (version 3.6.4, Beaverton, USA) and R package (version 3.3.3, Vienna, Austria). The normality of the data distribution was assessed by the Kolmogorov–Smirnov test. The Chi-square test was used to evaluate the significance of categorical variables, and the Mann–Whitney *U* test was performed to compare age differences between the training and validation sets. The significance level was set at p < 0.05 for all comparison tests. In the first task, FROC curves were applied to assess the sensitivity of the DL algorithm over FP rates for nodule identification in the validation and LUNA sets. Furthermore, to assess agreement in nodule identification between the reference standard given by experts and the DL algorithm, Bland–Altman (B–A) plots and 95% limits of agreement were applied. To further analyse the agreement between the DL algorithm and the experts for detecting micro-nodules or larger nodules, B–A analysis was also used in the subgroup analysis (nodular diameter < 4 mm or ≥ 4 mm). In the second task, the area under the ROC curve (AUC) was used to evaluate performance the DL algorithm for classifying positive/negative cases in the validation set and the LUNA set. The cut-off point of ROC curve was obtained by maximizing the Youden index. The corresponding sensitivity and specificity were plotted for the ROC curves. FROC scores or AUC values of the DL algorithm and their 95% confidence intervals (CIs) were obtained using the bootstrap method. Overall prevalence of NCPNs in three medical centers was calculated and stratified by age and gender. Density types, size and location distribution of PNs were analyzed and expressed with percentage.

## Result

### Training and validation of the DL algorithm

In total, 11,840,536 and 134,985 LDCT images obtained from a total of 39,014 imaging studies were assigned to the training set and validation set, respectively (Table 1). The mean age of patients included the training and validation set was 54.65 ± 15.91 years and 50.67 ± 13.52 years, respectively (p < 0.001). The female-male ratio was 42.7% to 57.3% in the training set and 47.1% to 52.9% in the validation set (p < 0.05). The nodule description for training and validation set showed in Table 2.

**Table 1:** Summarized results of performance assessments of DL algorithm and radiologists.

Data set	Rater	Time consumption per study (mins)	FROC score (95% CI)	ROC Sensitivity % (95% CI)	ROC Specificity % (95% CI)	AUC (95% CI)
LUNA (n = 888)	DL algorithm	0.08	0.80 [0.78, 0.82]	82 [73, 94]	82 [70, 91]	0.90 [0.88, 0.92]
Multi-centre validation (n = 582)	DL algorithm	0.09	0.75 [0.73, 0.78]	73 [63, 86]	85 [73, 96]	0.86 [0.84, 0.90]
	Radiologists	1.71* [0.87, 3.23]	NA^§^	83 [75, 89]	64 [51, 77]	0.73 [0.68, 0.78]

*Median, minutes range [min, max] for per-study time consumption in the multi-centre validation set.

^§^FROC score could not be calculated from individual radiologist.

**Table 2:** The nodule description for training and validation set.

Variables	Training (n = 38,414)	Validation (n = 582)	P-value
Total number of nodules	82,975 (100%)	1,265 (100%)	
**Size**			0.615
< 4 mm	11,534 (13.9%)	186 (14.7%)	
4-8 mm	51,113 (61.6%)	834 (65.9%)	
> 8 mm	20,328 (24.5%)	245 (19.4%)	
**Density**			0.208
SNs	63,642 (76.7%)	928 (73.4%)	
PSNs	2,406 (2.9%)	47 (3.7%)	
GGNs	16,927 (20.4%)	290 (22.9%)	
**Distribution**			0.217
RUL	21,822 (26.3%)	297 (23.5%)	
RML	15,267 (18.4%)	210 (16.6%)	
RLL	17,674 (21.3%)	267 (21.1%)	
LUL	13,857 (16.7%)	247 (19.5%)	
LLL	14,355 (17.3%)	244 (19.3%)	

*SNs* solid nodules, *PSNs* part-solid nodules, *GGN* ground glass nodules, *RUL* right upper lobe, *RML* right middle lobe, *RLL* right lower lobe, *LUL* left upper lobe, *LLL* left lower lobe.

The publicly available data (LUNA) included 226,589 test CT images from 888 studies, but no age or gender information was reported along due to anonymity of the patients’ private information. After 305 iterative learning epochs through the entire data set, the training process was stopped as no further improvement was seen in the loss of function of the algorithm.

### The diagnostic performance comparison

**Figure 4:**
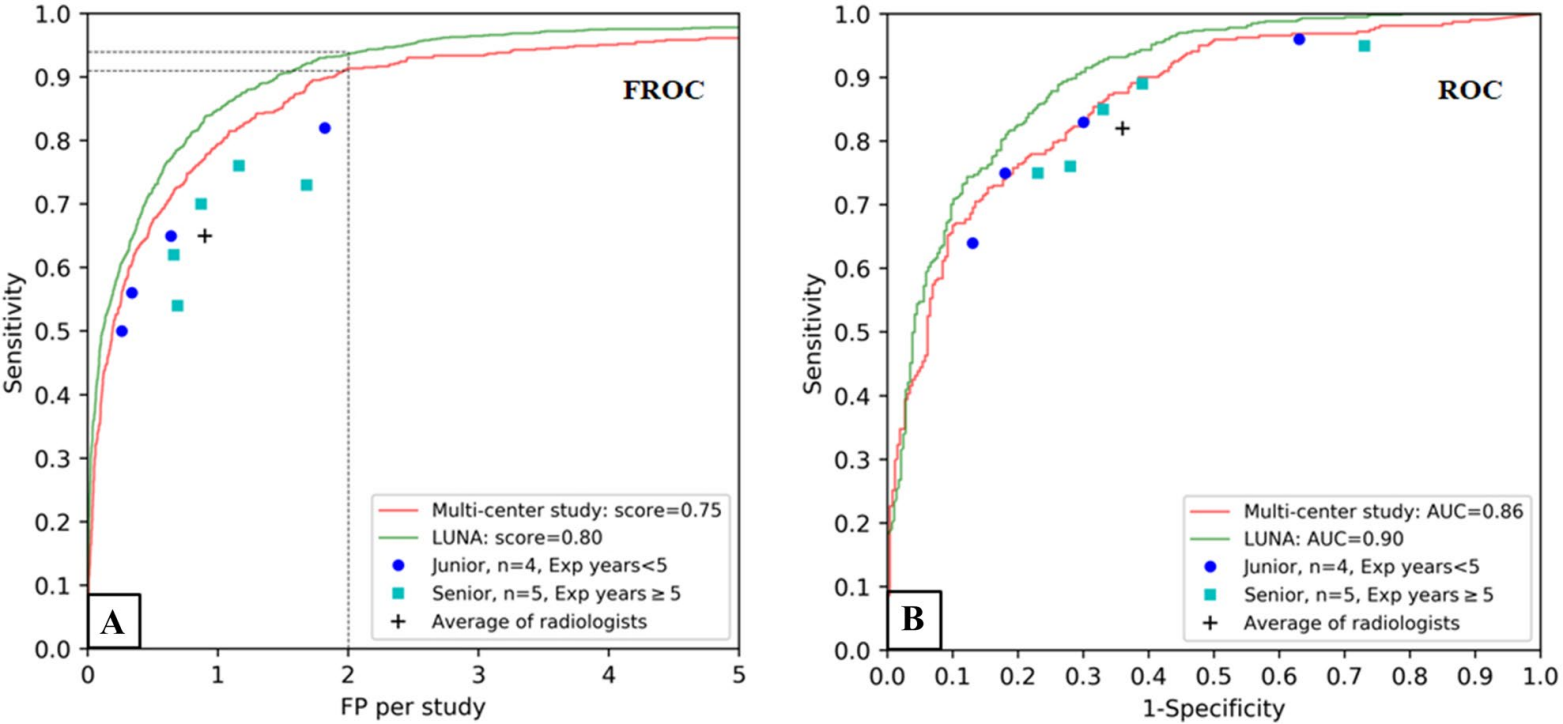
Performance assessment of the DL algorithm with FROC curves and ROC curves. Two assessment tasks, namely, nodule identification and classification for positive cases, are shown in the FROC and ROC curves, respectively. (**A**) FROC curves of the DL algorithm vs. individual or average performance of junior and senior radiologists assessed in the task of nodule identification. The DL algorithm outperformed all individual radiologists and the average radiologist performance in this task. (**B**) ROC curves of the DL algorithm vs. radiologists in the task of classification of positive cases. The performance of the algorithm was better than the average performance of the radiologists. The FROC scores and the AUC values showed slightly better performance of the algorithm on the LUNA test set than on the multi-centre validation set (FROC scores: 0.80 vs. 0.75; AUC values: 0.90 vs. 0.86). *FROC* freeresponse receiver operating characteristic, *ROC* receiver operating characteristic, *AUC* area under the curve, *FP* false positive.

The diagnostic performance of the DL model was compared to that of human raters, including junior and senior radiologists, and was assessed and displayed by FROC curves, AUC values, and the average duration of time to complete each study (Fig. 4, Table 1). The median duration of time to complete each study in the radiologist group was 1.71 min, which was significantly longer than that required by the DL model (0.09 min for the validation set and 0.08 min for the LUNA set). For the purposes of validation, two specific tasks, namely, nodule identification and classification of positive cases were assigned to the DL model and human raters in the multi-centre validation set. In addition, the same tasks were also performed using the DL model with the external test set (LUNA data). For the nodule identification task (Fig. 4A), the identification sensitivity over a range of FP rates per study obtained from the DL model outperformed all radiologists and their average identification sensitivity. The sensitivity of the DL model was more than 90% at a rate of 2 FP nodules identified per study (91% at 2 FPs/study), and the FROC score in the multi-centre validation set was 0.75 (95% CI 0.73–0.78). In comparison, the sensitivity of the DL model was more than 93.4% at a rate of 0.8FP nodules identified per study (93.4% at 0.8FPs/study) on the LUNA dataset, the FROC score for the LUNA data was 0.80 (95% CI 0.78–0.82), which was slightly better than the abovementioned score obtained in the multi-centre validation set. The sensitivity for the different nodule dimension in LUNA data with the DL algorithm were show in Table S1. The sensitivity for the different nodule sub-type in multi-centre validation set with the DL algorithm were show in Table S2.

For the positive-case classification task (Fig. 4B), in the multi-centre validation set, the DL model showed better performance than the average performance of the radiologists, the AUC of the DL model (AUC = 0.86, 95% CI 0.84–0.90) exceeded the average performance (AUC = 0.73, 95% CI 0.68–0.78) of the radiologists (p < 0.001), the sensitivity of the DL model (0.73, 95% CI 0.63–0.86) was lower than that of the radiologists (0.83, 95% CI 0.75–0.89), and the specificity of the DL model(0.85, 95% CI 0.73–0.96) was higher than of the radiologists(0.64, 95% CI 0.51–0.77) (Table 1); in the LUNA dataset, the AUC of the DL model achieved 0.90 (95% CI 0.88, 0.92), the sensitivity was 0.82 (95% CI 0.73–0.94) and the specificity was 0.82 (95% CI 0.70–0.91) (Table 1).

### The comparison between the reference standard and the DL model

**Figure 5:**
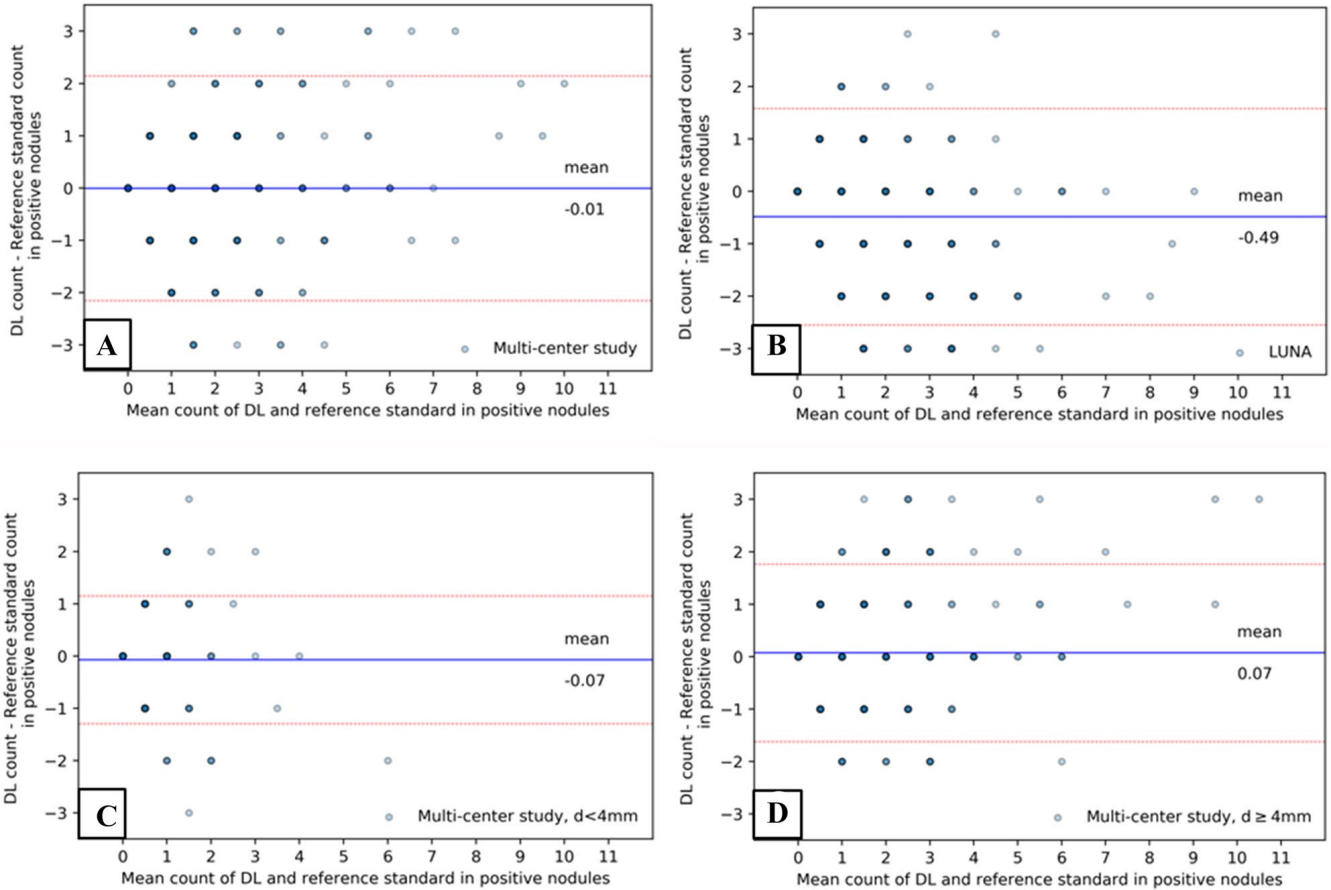
B–A plots showing agreement tests for positive nodule identification between the expert-reference standard and the DL algorithm. The plots elucidate the difference in the number of positive nodules identified by the experts and the DL algorithm (y-axis) over the mean of two measurements (x-axis) per study. For each plot, the line and value of the mean difference (blue line) and the upper and lower limits of 95% agreement (red dotted lines) are shown. The darker the dot colour was, the denser (more frequent) the data appeared on the specific X–Y axis area. (**A**) Agreement plot for the multi-centre validation set; (**B**) agreement plot for the LUNA set. To further assess the agreement for detecting micro-nodules and large nodules, subgroup analyses were performed for a nodule diameter < 4 mm (**C**) or ≥ 4 mm (**D**) in the multi-centre study data set.

B–A analysis was used to illustrate agreement in detecting positive nodules between the reference standard and the DL model on a per study level (Fig. 5). Good performance of the DL model in agreement with the reference standard would show a mean difference value/line close to 0. In our study, a mean agreement difference of − 0.01 (95% CI − 2.16 to 2.15) for the multi-centre validation set and − 0.49 (95% CI − 2.55 to 1.58) for LUNA test set were shown on the B-A plots (Fig. 5A,B). In the subgroup analyses of the multi-centre data, we demonstrated a mean agreement difference of − 0.07 (95% CI − 1.29 to 1.15) for a nodular diameter < 4 mm and 0.07 for a nodular diameter ≥ 4 mm (95% CI − 1.62 to 1.77) (Fig. 5C,D).

### Nodule based prevalence of NCPNs

**Figure 6:**
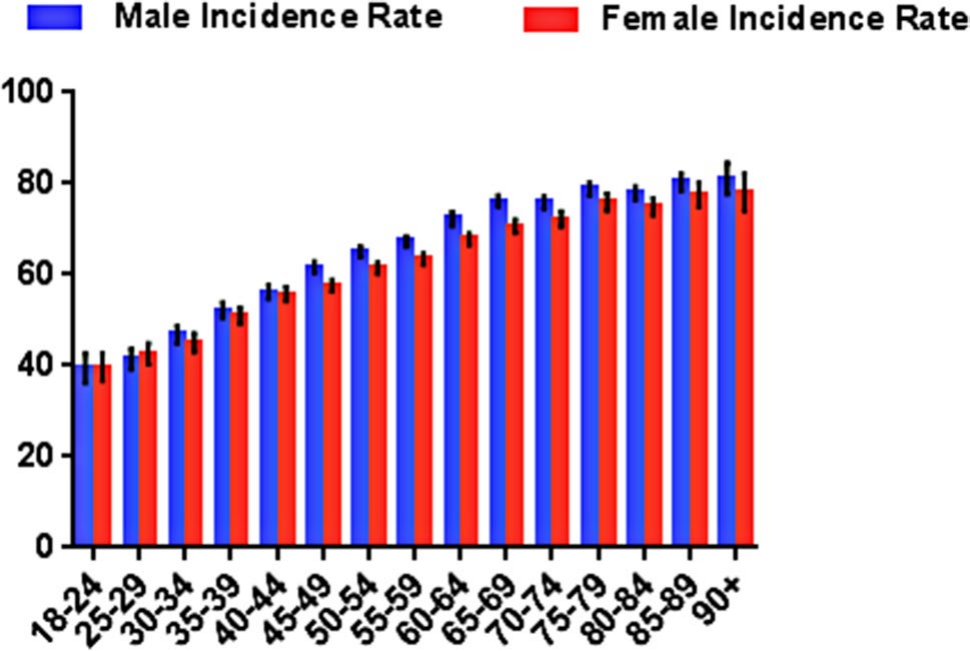
Age-specific rates of PNs identified by LDCT. Error bars represent the 95% confidence interval.

There were 64,168 cases included in our study to investigate the prevalence of NCPNs in China. Among them, 29,819 were females and 34,349 were males, with a median of 53 years ranging from 18 to 95 years. The age range of male is 18 to 95 years, the median age is 54. The age range of female is 18 to 95 years, the median age is 51. The age between males and females were not statistically significant (P > 0.05). A total of 139,325 nodules were found in 64,168 cases, there were 2.17 nodules/per patient. In 38,051 cases, at least one NCPN was observed. The prevalence of NCPNs was 59.3% (38,051/64,168, 95% CI 0.588–0.598). For male, the incidence was 61.7% (21,193/34,349, 95% CI 0.609–0.623), which was relatively higher than that for female 56.5% (16,858/29,819, 95% CI 0.551–0.57.6). The prevalence of NCPNs in men was consistently higher than that in women in nearly all age groups except those younger than 30 years (Fig. 6).

Among the 38,051 cases of NCPNs, 12,252 cases (32.2%) had single NCPNs, and 25,799 (67.8%) had multiple NCPNs. Incidence of NCPNs in men was significantly higher than that in women (P < 0.001). No matter for male or female, those with age 60–64 had the greatest number of NCPNs. The total number of NCPNs increased with the increase of age, peaked in age 60–64 and then dropped thereafter (Fig. S1). In 139,256 NCPNs, SNs, PSNs and GGNs accounted for 77.2%, 3.2% and 19.6%, respectively, there is a significant difference in the incidence among the three groups (P < 0.001). In regard to the location distribution, 24.2% were in right upper lobe (RUL), 16.6% in right middle lobe (RML), 21.0% in right lower lobe (RLL), 19.0% in left upper lobe (LUL), 19.2% in left lower lobe (LLL), there were also significant differences in the incidence of NCPNs between the lung lobes(P < 0.01). NCPNs with 4–8 mm were the most common size, which accounted for 61.3% of all NCPNs. Besides, 14.3% were 2–3 mm and 24.4% were 9–30 mm, the incidence of nodules of different sizes was also significantly different (P < 0.001). The proportion of nodular pattern, location and size in different sex and age subgroups was shown in Fig. S2.

## Discussion

In the current study, with the aid of DL model, we analyzed large-scale LDCT data from national multi-centre for the first time in China. Our results suggest that the method of initial DL model screening is feasible and efficient for large data analysis, and the incidence of pulmonary nodules has approached 60% in adults. One of the strengths of our study was that the DL-based neural network was trained with a large clinical dataset from three hospitals, indicating the generalizability of applying our algorithm. Compared with previous reports^13,14,23^ the major new findings of this study are as follows: First, our DL model showed higher identification sensitivity than all radiologists and their average sensitivity with a reasonably low diagnostic FP rate per case. Second, the DL model showed a better ROC-AUC performance and higher specificity than the average specificity of radiologists for classifying true positive cases. Third, this DL model trained with real-world clinical data demonstrated excellent agreement with radiology experts for detecting large and small lung nodules. Fourth, this DL model showed good performance in differentiating nodule dimensions and nodule sub-types.

FP reduction is a critical problem for lung nodule detection and influences DL model performance. Although some related experimental studies (non-clinically registered trials) have shown that DL models have a high detection rate, it remains questionable whether a medical DL system trained with a small dataset, such as LUNA/LIDC data, can prove its clinical practicability while still maintaining a low FP rate^14,22,40,41^. Built on a 50-layer deep neural network and trained with a large multi-centre database, our DL algorithm achieved a sensitivity of 91.0% at 2FPs/case, which was better than that reported in previous studies^42,43^. To better assess the performance of our DL model and prevent bias derived from the multi-centre study data, third-party data (LUNA dataset) were used as a external test set. In our study, the DL algorithm achieved a sensitivity of 93.4% at 0.8FPs/case based on the LUNA dataset. Cao et al.^44^ showed on their 3D-CNN network, the FP rate of each scan is 1, the corresponding sensitivity reached 90% on LUNA dataset, which is comparable to our DL model. Dou et al.^45^ used 3D CNNs for false positive reduction achieved a sensitivity of 90.7% at 4 FPs/case and Setio et al.^46^ Used multi-view convolutional networks (ConvNets) to extract the features achieved a sensitivity of 90.1% at 4 FPs/case, these two models were both slightly inferior than our DL model. The DL model showed an even better FROC score with the LUNA dataset than that with the multi-centre study data. The plausible reason is that LUNA data only included lung nodules larger than 3 mm in diameter as samples (we included nodules of all sizes), and nodules less than 3 mm in diameter and non-nodules were not counted, implying a higher sensitivity for detecting larger nodules. In LUNA dataset, the DL model showed good sensitivity both in nodules between 4–8 mm and > 8 mm, which indicated that the DL model was stable in differentiating nodule dimensions. Together, these results suggest the usefulness of our DL model design, namely, a classification neural network, for lowering the FP rate.

The DL algorithm performed with a high AUC for positive-case classification and exceeded the average performance of radiologists but had slightly worse performance on the LUNA data set. The mean sensitivity of radiologists reading independently has been reported to be 62.2–89.2% in previous literature^47^. In the present study, the average sensitivity of the nine radiologists was 64–96%, with an average of 82%. This study showed that the sensitivity of the DL algorithm was 73%, which was lower than the radiologists’ average sensitivity, but the specificity (85%) was higher than the mean specificity of the radiologists (64%).

Although there is a clear definition of lung nodules, inconsistencies between observers are common and more apparent in small nodules. There is moderate inter- and intra-observer agreement for nodule classification using the current recommendations for LDCT examinations of the chest^39,48^. The lack of a gold standard is an indisputable fact for lung nodule detection unless biopsy is provided (but is usually unavailable). Although a blinded review of all images was performed by the LIDC investigators, lesions missed by the original LIDC readers were also found^24,41^. In this study, we adopted the consensus of the three experts as the reference standard. The radiologists identified other lung nodules beyond the expert consensus, which were considered to be FP, resulting in increased sensitivity but reduced specificity.

The B–A analysis showed good agreement between the DL model and expert radiologists for detecting positive nodules, and subgroup analyses of micro-nodules and large nodules also demonstrated good agreement. This could be due to precise annotation and involvement of experienced raters in the training process. In this study, three expert radiologists were from three different hospitals and did not overlap with the previous training process. The high agreement indicates that the good performance of the DL model can be generalized. The DL model showed a large deviation in performance with the LUNA data set. This may not be a drawback of our DL model but rather may be due to different data sets from different countries with different raters and different nodule sizes.

Participating raters spent an average of 1.7 min to evaluate each case in the validation set. The rating time in this study was faster than that in usual clinical practice because the radiologists did not need to write diagnostic reports for these cases. They used a specially designed system for lung nodule assessment that did not require keyboard manipulation during the process. On the other hand, the DL model took only 5 s per case.

The prevalence of NCPNs in our study reached 59.3%, which was relatively higher than that reported in previous studies (13%–58%). There were some reasons for this increased percentage. First, the DL model is sensitive for detecting PNs although not all nodules are of clinical significance. Second, the DL model could find all nodules in several seconds, which can partly reduce the fatigue of doctors in finding nodules, which reduce the rate of missed diagnosis in radiologists. Third, the incidence of pulmonary nodules varies from race to race and the base population could also be different. Therefore, our results might be a little higher than those of previous studies.

In China, companies generally require employees to undergo an annual physical examination, which explains why young people do LDCT scanning and our study confirmed that the DL model was useful in the detection of PNs, which raise the working efficiency of radiologists. Few literatures have reported the feasibility of the DL model in big data analysis although some previous studies applied big data technology to predict readmission risk for congestive heart failure or for post-cystectomy participants^23,24^. To the best of our knowledge, our study was the first large-scale one to use the DL model for detection PNs. Without the DL model, we would not be able to get so much data and radiologists would not be able to identify all nodules in a very short time.

Demographic factors were associated with the occurrence of NCPNs. Increasing age was strongly associated with NCPNs, which was consistent with previous reports^29,31^. In addition, there was a significant difference in the distribution of NCPNs between males and females. Prior studies have shown that in persons younger than 70, the PN was slightly more commonly seen among women than men. However, in our study, men had a higher incidence of PN in nearly all age groups except for those younger than 30 years old, possibly due to other related precipitating factors of PNs in males such as smoking history. Our study confirmed that the method of the DL model screening is feasible.

However, there are still several limitations to this study. First, we must admit that there was no gold standard but only a reference standard of lung nodule detection for this study, even for the LUNA data. Although we adopted the reference standard of an assessment by three highly experienced radiologists with more than 20 years of experience, some lung nodules might have been omitted by experts due to human error. Second, both our study data and the LUNA data lacked some baseline information, such as smoking history, lung diseases and other related morbidities, which can further help in the management of lung cancer screening^27^. Third, although, the location and morphology of lung nodules may also act as predictors of lung cancer^49^, we did not further analyze the incidence of lung cancer screened by the DL model. There is no reference standard in the LUNA dataset to distinguish semi-solid nodules and ground glass nodules, so our model does not compare the performance of nodules sub-types in LUNA dataset, which can be carried out in future studies. However, in the multi-centre validation set, the DL model showed good performance in the classification of nodule sub-types.

In summary, this was a clinically registered big data screening study with a blinded review design that compared the performance of a DL system trained with LDCT cases and supervised by radiologists with the performance of the doctors themselves for lung nodule identification, and to investigate the prevalence of NCPNs in China. The results showed that the DL system had better identification sensitivity and performance than the average sensitivity and performance of the radiologists. In addition, this model was highly consistent with the expert radiologists in terms of lung nodule identification, regardless of nodule size. With the merits of good performance, fast processing and efficiency, the application of DL may be possible for clinical screening tasks and can serve as a radiologist's assistant. With the help of AI, the big data analysis shows a high prevalence of NCPNs in LDCT screening. The right upper lobe is the most common location of nodule distribution. Most of the detected PNs are solid nodules, and their size is between 5 and 8 mm.

## Supplementary information

Supplementary Information.

## Data Availability

The authors declare that they had full access to all of the data in this study and the authors take complete responsibility for the integrity of the data and the accuracy of the data analysis.
