# Supplementary material for: Development and clinical application of deep learning model for lung nodules screening on CT images
Source: Sci Rep. 2020 Aug 12;10:13657. doi: 10.1038/s41598-020-70629-3 (PMC7423892; doi:10.1038/s41598-020-70629-3)
Supplement: Supplementary file 1 — Supplementary Information. [file 41598_2020_70629_MOESM1_ESM.docx]

**Development and Clinical Application of Deep Learning model for Lung Nodules Screening on CT Images**

SiJia Cui^1,2^, Shuai Ming^1^, Yi Lin^1^, FangHong Chen ^1^, Qiang Shen ^1^, Hui Li^3^, Gen Chen^3^, XiangYang Gong^1,4*^ and HaoChu Wang^1*^

^1^Department of Radiology, Zhejiang Provincial People’s Hospital, Afﬁliated People’s Hospital of Hangzhou Medical College, 310013, Hangzhou, China

^2^The Second Clinical Medical College, Zhejiang Chinese Medical University, 310053, Hangzhou, China

^3^Hangzhou Yitu Healthcare Technology Co., Ltd, 310000, Hangzhou, China

^4^Institute of Artiﬁcial Intelligence and Remote Imaging, Hangzhou Medical College, 310000, Hangzhou, China

*These authors contributed equally to this work

*Correspondence to:* Haochu Wang(wanghcbox@163.com) and XiangYang Gong(cjr.gxy@hotmail.com). Department of Radiology, Zhejiang Provincial People’s Hospital, Affiliated People’s Hospital of Hangzhou Medical College, Institution of Teleradiology and Artificial Intelligence of Hangzhou Medical College, Hangzhou 310014, China.

**Methods**

**Lung CT protocol in multi-centre study**

The multi-centre study was performed at three screening sites to minimize the data bias from a single centre. The chest CT images were acquired with a low-dose CT protocol on 64-slice multi-detector CT scanners (Somatom Definition AS, AS^+^ & Flash, Siemens, Germany; Optima 680, Discovery 750 HD, GE Healthcare, USA). The following CT protocol settings were implemented: spiral CT scanning; 120 kVp; automatic modulation of 3D radiation dose (‘Smart mA’ + organ dose modulation) with lower bound 100 mA, maximal bound 200 mA and noise index 10; rotation time: 0.35 s ; modulation 35–70 mAs; pitch=0.992:1 and collimation: 80 mm. The radiation dose, volume CT dose index (CTDIvol) and dose length product (DLP)=CTDIvol × length of exposure) may vary depending on patient attenuation and length of the acquisition. The DLP is between 70 and 200 mGy.cm (0.98 mSv to 2.8 mSv) (the effective dose is calculated by multiplying DLP by a thoracic conversion factor of 0.01421), for an average DLP of 100 mGy.cm. For every patient, CTDIvol and DLP are recorded. Effective dose and size-specific dose estimates will be then calculated. Concerning the additional radiation for included patients, our CT protocol has an expected effective dose between 0.10 mSv and 0.20 mSv. Breath-holding lasted for 15 seconds to prevent image motion artefacts during the scan. The lung LDCT images from the training and validation sets were then reconstructed at a slice thickness of 1-2 mm with a spacing interval of 2 mm. All image readings were performed at a lung window width of 1500 HU and a window level of - 400 HU. Images with severe artefacts and missing clinical reports were excluded from the study.

**AI algorithm**

The algorithm model was implemented using the TensorFlow library (Version 1.8.0, Google, Mountain View, USA, https://www.tensorflow.org) and was executed on a GeForce GTX 1080Ti GPU (NVidia, Santa Clara, USA), which was used for data training and validation. The learning model was optimized by stochastic gradient descent (SGD) with a back-propagation method at a learning rate of 0.01 (also known as the fine-tuning process), iteratively minimizing the error (i.e., loss function) between the model output and ground truth (i.e., reference standard given by the expert radiologists). Batch normalization was used for regularization in each convolutional layer.

**Statistical Analysis**

The x-axis of the FROC curve was defined as six FP rates per study (FPs per study), ranging from 0 to 5 FP nodules per study. The y-axis of the FROC curve was the true positive fraction of all nodule readings (i.e., sensitivity).

A B-A plot was created to show the difference in the number of positive nodules between the experts and the DL algorithm (y-axis) over the mean of the two measurements (x-axis) per study.

AUC comparisons were performed using the method described by Hillis et al. The individual performance of the human reviewers, including junior and senior radiologists, was displayed as a standalone point on the FROC and ROC curves, and the average performance of all radiologists was also calculated to compare with that of the DL algorithm on these curves.

**Result**

| **Table S1: Sensitivity of the DL algorithm for the different nodule dimension in LUNA Dataset** | | |
| --- | --- | --- |
| Different nodule dimension | Sensitivity % | 95% CI |
| 4-8 mm | 91.1 | [86.3,95.4] |
| >8mm | 95.7 | [91.7,99.5] |
| 95% CI: 95% confidence interval | | |

| **Table S2: Sensitivity of the DL algorithm for the different nodule sub-types in multi-center validation set** | | |
| --- | --- | --- |
| Different nodule sub-types | Sensitivity % | 95% CI |
| Solid | **93.3** | [89.2,96.9] |
| Sub-solid | 90.5 | [86.7,95.5] |
| GGN | 91.7 | [87.4,96.1] |
| GGN: ground glass nodules; 95% CI: 95% confidence interval | | |

**
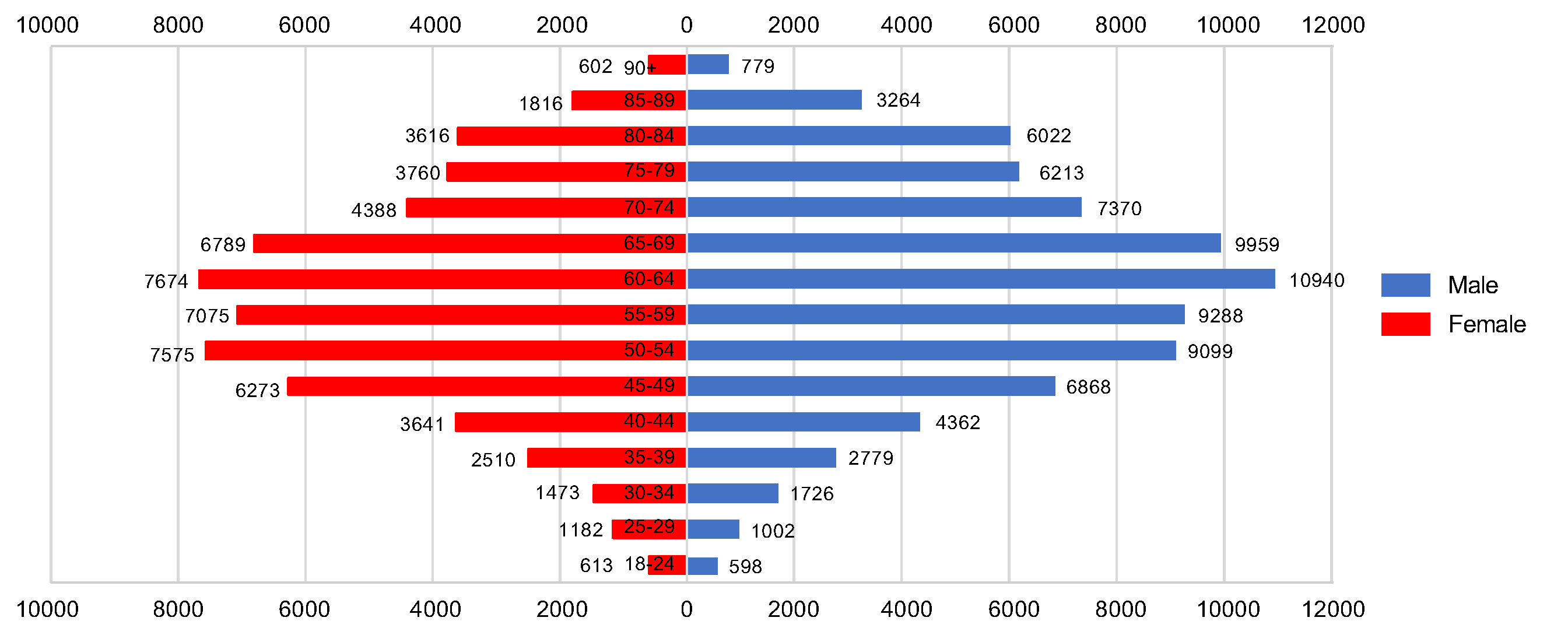
**

**Figure S1** Number of pulmonary nodules in different age and gender subgroups

**
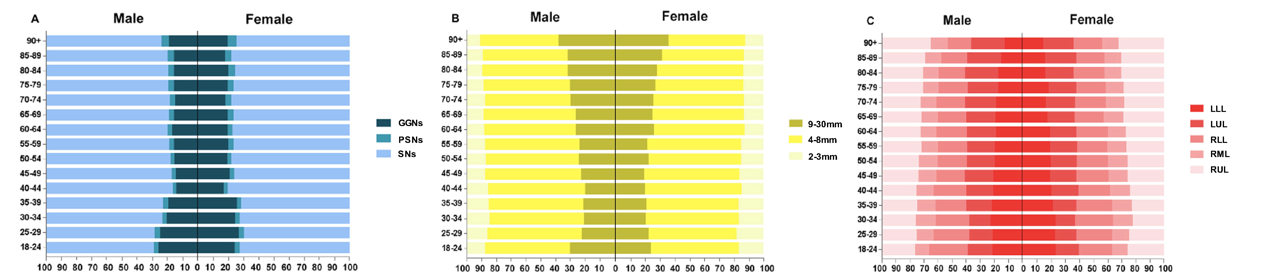
**

**Figure S2**. Proportion of different types and locations of PNs categorized by age and gender. The X-axis represents the percentage, the Y-axis represents the age, and the two sides of the sub-coordinate axis represent the percentage of different nodules in different genders. SNs, solid nodules; PSNs, part-solid nodules; GGNs, ground glass nodules; RUL, right upper lobe of lung; RML, right middle lobe of lung; RLL, right lower lobe of lung; LUL, left upper lobe of lung; LLL, left lower lobe of lung.
